# South Carolina and Dr. Clark

**Published:** 1851-10

**Authors:** 


*South Carolina and Dr. Clark. Complimentary to the Profession.*—To prove that medical men do not always go unrewarded by those to whom important services may have been rendered, we give the following instance as an exception. The State of South Carolina has recently presented to her distinguished son, Dr. C. J. Clark, a gold medal, weighing two and a half oz., upon one side of which is a representation of the landing of Vera Cruz; and on the other, the South Carolina "Coat of Arms." This honor has been conferred in consideration of the valuable services rendered by Dr. Clark to her brave sons who fought, and many of whom fell, upon the bloody plains of Mexico, in maintaining the honor of the State and in defending our common country. South Carolina knows how to encourage talent, and to reward true merit — and in this she has given us many noble examples worthy of her distinguished name.

We hope Dr. Clark will not feel envious when he hears in what manner we were rewarded for three months' services on the Rio Bravo, Mexico. Ours came from head-quarters —Washington city. In turning over to the Quarter-master at this place the medicine and hospital stores in our charge, one or two silver-washed catheters were missing; these were promptly reported to head-quarters, and in due time a bill of $40 was returned to us, claiming indemnity for that which was loaned and used up in the service of the country.

From that day to this our thirst for military glory has been on the wane, and we wish henceforward to be regarded as an uncompromising advocate for the "Peace Congress."— *New Orleans Med. and Surg. Journal.*

